# GPR55 Deletion in Mice Leads to Age-Related Ventricular Dysfunction and Impaired Adrenoceptor-Mediated Inotropic Responses

**DOI:** 10.1371/journal.pone.0108999

**Published:** 2014-10-02

**Authors:** Sarah K. Walsh, Emma E. Hector, Anne-Christine Andréasson, Ann-Cathrine Jönsson-Rylander, Cherry L. Wainwright

**Affiliations:** 1 Institute for Health & Wellbeing Research, Robert Gordon University, Riverside East, Aberdeen, United Kingdom; 2 AstraZeneca R&D, IMED CVGI, Bioscience, Mölndal, Sweden; Emory University, United States of America

## Abstract

G protein coupled receptor 55 (GPR55) is expressed throughout the body, and although its exact physiological function is unknown, studies have suggested a role in the cardiovascular system. In particular, GPR55 has been proposed as mediating the haemodynamic effects of a number of atypical cannabinoid ligands; however this data is conflicting. Thus, given the incongruous nature of our understanding of the GPR55 receptor and the relative paucity of literature regarding its role in cardiovascular physiology, this study was carried out to examine the influence of GPR55 on cardiac function. Cardiac function was assessed via pressure volume loop analysis, and cardiac morphology/composition assessed via histological staining, in both wild-type (WT) and GPR55 knockout (GPR55^−/−^) mice. Pressure volume loop analysis revealed that basal cardiac function was similar in young WT and GPR55^−/−^ mice. In contrast, mature GPR55^−/−^ mice were characterised by both significant ventricular remodelling (reduced left ventricular wall thickness and increased collagen deposition) and systolic dysfunction when compared to age-matched WT mice. In particular, the load-dependent parameter, ejection fraction, and the load-independent indices, end-systolic pressure-volume relationship (ESPVR) and *E*_max_, were all significantly (*P*<0.05) attenuated in mature GPR55^−/−^ mice. Furthermore, GPR55^−/−^ mice at all ages were characterised by a reduced contractile reserve. Our findings demonstrate that mice deficient in GPR55 exhibit maladaptive adrenergic signalling, as evidenced by the reduced contractile reserve. Furthermore, with age these mice are characterised by both significant adverse ventricular remodelling and systolic dysfunction. Taken together, this may suggest a role for GPR55 in the control of adrenergic signalling in the heart and potentially a role for this receptor in the pathogenesis of heart failure.

## Introduction

G protein coupled receptor 55 (GPR55) belongs to a group of rhodopsin-like seven transmembrane/g-protein coupled receptors and was originally isolated in human striatum [1]. GPR55 has since been shown to be widely distributed in a variety of cell types and in the central nervous, gastrointestinal, and cardiovascular systems, in both humans [2] and rodents [3]. The downstream signalling mechanisms following activation of GPR55 remain unclear although activation of Gα_q/11_ or Gα13 culminating in an eventual elevation of intracellular calcium (Ca^2+^) and phosphorylation of extracellular signal-regulated kinase (ERK) and/or nuclear factor of activated T cells (NFAT) has been implicated [4], [5], [6]. Furthermore, while the exact physiological/pathophysiological function of GPR55 remains to be determined, studies have suggested a role in pain, bone development, carcinogenesis, pregnancy, metabolism (reviewed by [2]), and finally in the control of cardiac haemodynamics.

In terms of the cardiovascular system, a role for GPR55 was proposed on the basis of accumulating evidence from a series of studies investigating the profound haemodynamic (hypotension and bradycardia) effects of cannabinoid ligands, which were initially believed to be mediated primarily through the classic cannabinoid receptors, CB_1_ and CB_2_, (reviewed by [7]). However, combined evidence from studies using mice deficient in either CB_1_ or CB_2_ and from experiments employing various pharmacological agonist/antagonist combinations, have revealed that many cannabinoid-induced haemodynamic responses are mediated by non-CB_1_/CB_2_ receptors [8], [9]. Moreover, cannabinoids that have little or no affinity for the CB_1_/CB_2_ receptors have also been shown to exert cardiovascular effects, further suggesting a role for additional receptor(s) in mediating these effects [10], [11]. Based on the findings that some vasoactive cannabinoids (e.g. abnormal cannabidiol and O-1602) are potent agonists of GPR55 [3], [5], [12], [13], the latter has been proposed as a possible third cannabinoid receptor [14], [15]. However, a more recent study investigating an array of cannabinoids as possible ligands for GPR55 demonstrated that only lysophosphatidylinositol (LPI), rimonabant, and AM251 are agonists for this receptor, and that neither abnormal cannabidiol nor O-1602 activate GPR55 [16]. Thus, given the incongruous nature of our understanding of the GPR55 receptor and the relative paucity of literature regarding its role in cardiovascular physiology we conducted a study using the previously described homozygous GPR55-deficient (GPR55^−/−^) mouse [12], [17], to examine the influence of GPR55 on cardiac physiology/function (assessed via pressure volume loop analysis).

## Methods

### Breeding and genotyping of GPR55 mice

Heterozygous GPR55 knockout mice were intermated to produce F1 mice homozygous for the GPR55 mutation (GPR55^−/−^) and wild-type (WT) littermate controls and genotyped as previously described [12]. Both male and female WT and GPR55^−/−^ were bred and housed in the University of Aberdeen Medical Research Facility. Animals were maintained at a temperature of 21±2°C, with a 12 h light/dark cycle and with free access to food and tap water. Animals were obtained on a daily basis and allowed to acclimatize before commencing the study. All studies were performed under an appropriate Project License authorized under the UK Animals (Scientific Procedures) Act 1986. All *in vivo* work is reported in accordance with the ARRIVE guidelines [18].

### Measurement of ventricular function

Mice were anaesthetised with a mixture of ketamine (120 mg kg^−1^; Vetalar, Pfizer, Dublin, Ireland) and xylazine (16 mg kg^−1^; Rompun, Bayer, Dublin, Ireland) via intraperitoneal (i.p.) injection and the trachea cannulated to allow artificial respiration when required. The right jugular vein was cannulated with flame-stretched Portex polythene tubing (0.58 mm ID×0.96 mm OD; Smiths Medical International Ltd., Hyde, Kent, UK) for drug administration and the mice ventilated on room air (130–140 strokes min^−1^ and tidal volume 150–210 µL calculated based on individual animal weight; Harvard small animal respiration pump; Edenbridge, Kent, UK). Ventricular function was measured in mice via pressure volume analysis using a method adapted from Pacher et al. [19]. Briefly, the chest was opened, the pericardium removed, the apex of the left ventricle punctured with a 27 g needle, and a 1.4-Fr pressure conductance catheter (SPR-839; Millar Instruments, Houston, Texas, US) inserted into the ventricle to record cardiac function via the MPVS-Ultra Single Segment Foundation System (Millar Instruments, US). A steel thermistor probe (Fisher Scientific Ltd., Loughborough, Leicestershire, UK) was inserted into the rectum to measure core temperature, which was maintained at 37–38°C with the aid of a Vetcare heated pad (Harvard Apparatus Ltd.). Anaesthesia was maintained throughout by administration of 50 µl 25 g^−1^ (b.w.) of the ketamine and xylazine mixture via i.p. injection every 40 min or as required. After a stabilisation period of approximately 20 min baseline cardiac function was recorded and then a bolus dose of dobutamine (10 µg kg^−1^) administered to mice to examine contractile reserve. To obtain measurements of load-independent contractility (time varying elastance; *E*_max_) and the slopes of both the end-systolic pressure-volume relationship (ESPVR) and end-diastolic pressure-volume relationship (EDPVR), venous return (left ventricular preload) was varied via transient occlusion of the inferior vena cava. Finally, to enable correction of pressure volume loops during data analysis the parallel conductance (Vp) was calculated via the administration of a small volume of hypertonic saline (15%; i.v.) to mice. Following completion of the *in vivo* protocol, animals were euthanised via an overdose of anaesthetic and blood collected to allow volume calibration of the catheter using heparinized blood-filled calibration cuvettes. Finally, the heart was removed and the ventricular tissue fixed in 10% formal buffered saline for histological studies.

### In vivo experimental protocols

Young male/female (10 week old) WT (n = 15; 8 males & 7 females) and GPR55^−/−^ (n = 15; 8 males & 7 females) mice were used to investigate the role of GPR55 in the control of basal cardiac function. As preliminary data had demonstrated that 8 month old GPR55^−/−^ mice had elevated blood pressure compared to WT mice (unpublished findings from AstraZeneca) an additional series of experiments was carried out using mature mice (8 months old; WT (n = 14; 7 males & 7 females) and GPR55^−/−^ (n = 14; 7 males & 7 females)) to investigate whether any observed changes in cardiac function were influenced by advancing age. As there were no gender-related differences in either cardiac function or structure the data presented represents the pooled data from both males and females within each group.

### Histological assessment of cardiac morphology

For haemotoxylin and eosin (H&E) staining, fixed ventricular tissue was embedded in paraffin wax (Thermo Scientific) and 5 µm sections cut. Sections were dehydrated through a series of histosolve (Thermo Scientific) and graded alcohols and incubated in haematoxylin to stain nuclei, and subsequently incubated in 0.5% acid alcohol, Scott's tap water substitute, and finally eosin to stain the remaining cellular material. After staining, sections were mounted with a xylene substitute mountant (Thermo Scientific) and covered with a cover slip. Analysis of the tissue was carried out with the use of a Leica DMLB light microscope (Leica Microsystems, Milton Keynes, Bucks, UK) at a magnification of ×25 for gross morphological measurements and ×400 for cardiomyocyte analysis and nuclei quantification. For gross morphology, multiple measurements of the right ventricular free wall, the interventricular septal wall, and the left ventricular free wall were made using computerised planimetry (ImageJ software, National Institute of Health (NIH), Rockville Pike Bethesda, MD). In addition, left ventricular chamber area was calculated as a percentage of total left ventricle area. For cardiomyocyte measurements, the cross-sectional area of 5–10 cardiomyocytes with a centrally located nucleus and circular shaped cell membrane was measured in the left ventricular free wall of each animal using ImageJ. Finally, to detect any changes in myocardial cellular populations, the number of positively stained nuclei within 10 fields randomly selected from the left ventricle was quantified.

### Quantification of cardiac collagen deposition

For Masson Trichrome (MT) staining, paraffin embedded ventricular sections (5 µm) were dehydrated through a series of histosolve (Thermo Scientific) and graded alcohols and incubated in biebrich scarlet-acid fuchsin solution to stain cellular material, and subsequently incubated in a phosphomolybdic–phosphotungstic acid solution, aniline blue, and finally 1% acetic acid to stain the collagen fibers. After staining, sections were mounted with a xylene substitute mountant (Thermo Scientific) and covered with a cover slip. Photomicrographs (×400) were taken with the use of a Leica DMLB light microscope (Leica Microsystems, Milton Keynes, Bucks, UK) and collagen volume fraction (CVF) was calculated by determining the percentage area of blue (collagen) stained tissue within 10 fields randomly selected from the left ventricle using computerised planimetry (ImageJ software, National Institute of Health (NIH), Rockville Pike Bethesda, MD). Briefly, images were converted to RGB stacks, separated into a montage, and the red channel thresholded to detect the stained collagen (http://rsbweb.nih.gov/ij/docs/examples/stained-sections/index.html). For continuity, the same threshold was used on images from all animals and the measurement of collagen restricted to the left ventricle to correspond with the functional pressure volume loop data.

### Immunostaining for GPR55

Immunohistochemical staining for GPR55 was performed in fixed heart sections from all groups. Paraffin embedded myocardial tissue sections were cut (4 µm) and mounted onto polysine coated slides. Following dehydration and deparaffinization, antigen retrieval was performed by pressure cooking using Diva (Biocare, USA). Immunohistochemical staining was carried out using the Intellipath Immunostainer (Biocare) and involved the following steps. Endogenous peroxidase activity was blocked via incubation with Peroxidize 1 (Biocare, USA), followed by Background sniper (Biocare, USA). The primary antibody, GRP55-A162T750 (MBL International Corporation) was diluted 1∶500 using DaVinci Green Diluent (Biocare, USA) and incubated on the slides for 1 hour. Sections were then incubated with a two-step biotin free micro-polymer detection system (MACH3; Biocare, USA), counterstained with Tachas hematoxylin prior to incubation with 3,3′-diaminobenzidine (Betazoid DAB, Biocare, USA). Sections were then finally dehydrated and cleared through a series of graded alcohols and xylene and mounted in Mountex mounting medium (HistoLAb AB, Sweden). Two negative control methods were used to verify the specificity of the antibody reaction. The first negative control was performed by replacing the MACH3 system with buffer to exclude unspecific binding of the secondary antibody to the tissue. Secondly, a dilution test was performed, where the primary antibody was diluted to a concentration that did not give rise to positive staining. Photomicrographs of sections were taken at a magnification of ×200 with the use of a Sony progressive 3CCD colour video camera.

### Solutions and chemicals

All chemicals were purchased from either Sigma-Aldrich (Dorset, UK) or Fisher Scientific UK Ltd. (Loughborough, UK) unless otherwise stated. Scott's tap water substitute contained (in mM): 81 MgSO_4_.7H_2_O and 42 NaHCO_3_.

### Statistical analysis

For haemodynamic and morphological data a one-way ANOVA and bonferroni post-hoc test was used to compare selected experimental groups. All data was expressed as the mean±s.e.m and significance was determined as *P*<0.05.

## Results

### Effect of gene deletion for GPR55 on baseline cardiac function in young mice

**Figure 1:**
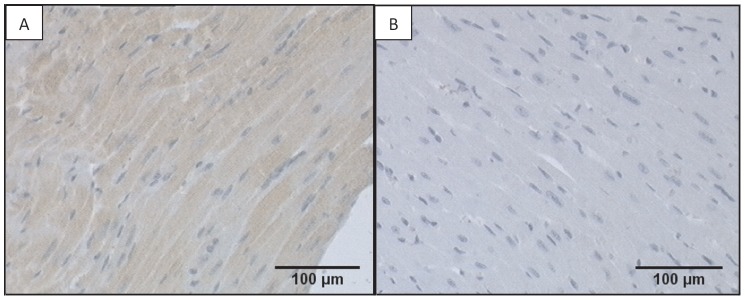
Expression of GPR55 in ventricular tissue from WT and GPR55^−/−^ mice. Photomicrographs taken at ×200 demonstrate positive staining for GPR55 in ventricular tissue (localised to the cardiomyocytes) from WT mice (A), but not GPR55^−/−^ mice (B).

**Figure 2:**
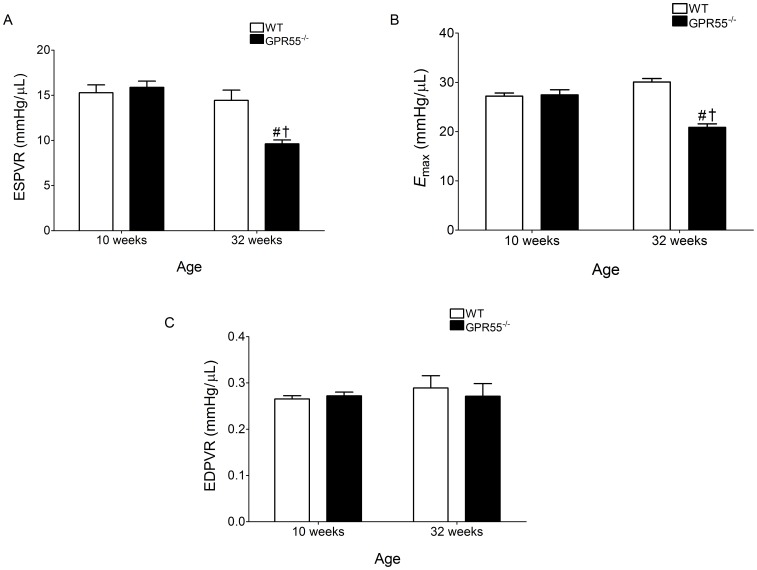
Load-independent (ESPVR, EDPVR, and *E*_max_) haemodynamic parameters in both WT and GPR55^−/−^ mice. In mature mice with a genetic deletion for GPR55 (GPR55^−/−^), baseline systolic function, but not diastolic function, was adversely affected. In particular, significant reductions in both ESPVR (A) and *E*_max_ (B) indicate attenuated cardiac contractility, while EDPVR (indicative of relaxation rate) was unaffected (C) in mature GPR55^−/−^ mice. Data is expressed as mean±s.e.m. (n = 14–15) *P<0.05 vs. WT (Young); #P<0.05 vs. GPR55^−/−^ (Young); †P<0.05 vs. WT (Mature).

Positive staining for GPR55 was detected in ventricular tissue from WT (Figure 1A) but not GPR55^−/−^ mice (Figure 1B). Furthermore, image analysis revealed diffuse staining for GPR55 throughout the cardiomyocytes, suggestive of a role for this receptor in the control of contractile function. However, pressure volume loop analysis revealed that, with the exception of heart rate (HR), which was significantly elevated in 10 week old GPR55^−/−^ mice, none of the other load-dependent indices of left ventricular (LV) systolic and diastolic function; end systolic pressure (ESP), end systolic volume (ESV), stroke volume (SV), cardiac output (CO), ejection fraction (EF), maximum (d*P*/d*t*_max_) and minimum (d*P*/d*t*_min_) derivatives of pressure, arterial elastance (*E*_a_; an index of LV afterload and reflective of peripheral vascular resistance), end diastolic pressure (EDP) and volume (EDV), recorded in this study differed significantly when compared to 10 week old WT mice (Table 1). Furthermore, none of the load-independent measurements of LV function, ESPVR, EDPVR, and *E*_max_, differed significantly between both groups of mice (Figure 2).

**Table 1:** Load-dependent haemodynamic parameters in control (WT) mice and those with a genetic deletion for GPR55 (GPR55^−/−^).

	Young Mice (10 week old)		Mature Mice (8 month old)	
	WT (n = 15)	GPR55^−/−^ (n = 15)	WT (n = 14)	GPR55^−/−^ (n = 14)
Body weight (g)	21±0.5	23±0.5	30.5±0.5	30.7±0.7
HR (BPM)	395±7	420±7*	406±8	425±10
ESP (mmHg)	91.8±2.2	93.4±3.5	111.8±3.5*	101.9±3.7
EDP (mmHg)	4.5±0.4	5.1±0.3	5.2±0.3	5.3±0.3
ESV (µL)	11.9±0.4	13.2±0.8	16.2±0.7*	22.7±0.7# †
EDV (µL)	21.8±0.8	23.1±0.6	29.3±0.8*	35.1±0.9# †
SV (µL)	11.2±0.4	11.3±0.5	15.1±0.8*	14.2±0.8#
SW (mmHg*µL)	910±76	939±66	1377±67*	1278±103
CO (µL/min)	4430±214	4768±265	6092±382*	5994±353
*E*_a_ (mmHg/µL)	7.3±0.7	8.1±0.6	8.1±0.5	6.7±0.4
EF (%)	51.7±1.9	50.9±1.7	51.5±2	40.1±1.6# †
d*P*/dt_max_ (mmHg/s)	7800±339	7800±486	8760±563	8399±457
d*P*/dt_min_ (mmHg/s)	−7773±452	−7290±453	−8448±578	−8643±714

Cardiac function is unaffected by age in WT mice, but undergoes a significant deterioration in GPR55 deficient mice. Data is expressed as mean±s.e.m. (n = 14–15).

*P<0.05 vs. WT (Young);

# P<0.05 vs. GPR55^−/−^ (Young);

† P<0.05 vs. WT (Mature).

### Age dependent changes of GPR55 gene deletion on baseline cardiac function

**Figure 3:**
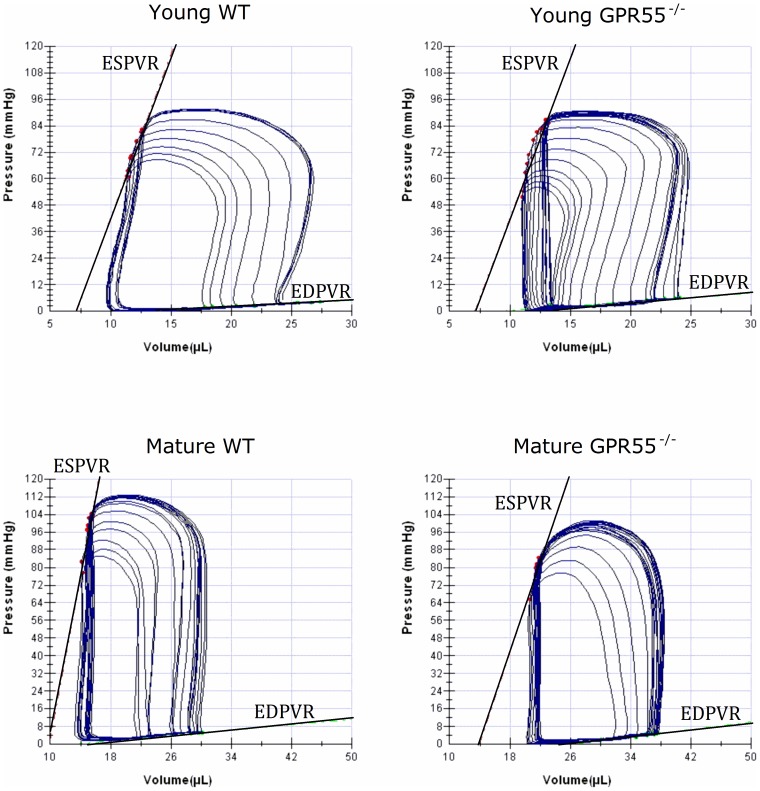
Representative left ventricular pressure volume loops from all experimental groups are included and illustrate the emerging systolic dysfunction (i.e. downward and rightward shift in the ESPVR curve) associated with mature GPR55^−/−^ mice.

In terms of load-dependent cardiovascular variables, both indices of systolic and diastolic function were significantly altered with age in both WT and GPR55^−/−^ mice (Table 1). In particular, ESP, ESV, EDV, SV, SW, and CO were all significantly elevated in mature WT mice compared with young mice (Table 1), which in part may be due to the increased circulating blood volume in these larger animals. In contrast, only SV, EDV, and ESV were significantly increased in mature GPR55^−/−^ mice, and in terms of the latter two indices they were also significantly elevated in comparison to age-matched WT mice. Furthermore, mature GPR55^−/−^ mice also exhibited compromised systolic function as EF was significantly decreased in these mice when compared to both young GPR55^−/−^ mice and age-matched WT controls (*P*<0.001; Table 1). This emerging systolic dysfunction appeared to be due to the significant increase in EDV (*P*<0.001) recorded in the mature GPR55^−/−^ mice, which was not accompanied by a sufficient increase in SV (to maintain EF), when compared to age-matched WT controls (Table 1). Furthermore, load-independent measurements obtained during transient occlusion of the inferior vena cava (to alter preload), demonstrated a significant downward shift in both the ESPVR slope (*P*<0.001; Figure 2A) and the time varying elastance (*E*_max_; *P*<0.0001; Figure 2B) in the mature GPR55^−/−^ mice indicative of decreased contractility/inotropy. In contrast, the slope of the EDPVR (indicative of an increase in LV chamber stiffness; Figure 2C) did not differ significantly between any of the experimental groups examined and thus load-independent diastolic function did not appear to be altered following deletion of the GPR55 gene. Taken together, the changes in both load-dependent and load-independent indices of cardiac function observed in the mature GPR55^−/−^ mice suggest that the emerging systolic dysfunction appears to be due to the deleterious combination of both GPR55 gene deletion and advancing age in these animals (representative pressure volume loops of this cardiac dysfunction are illustrated in Figure 3).

**Figure 4:**
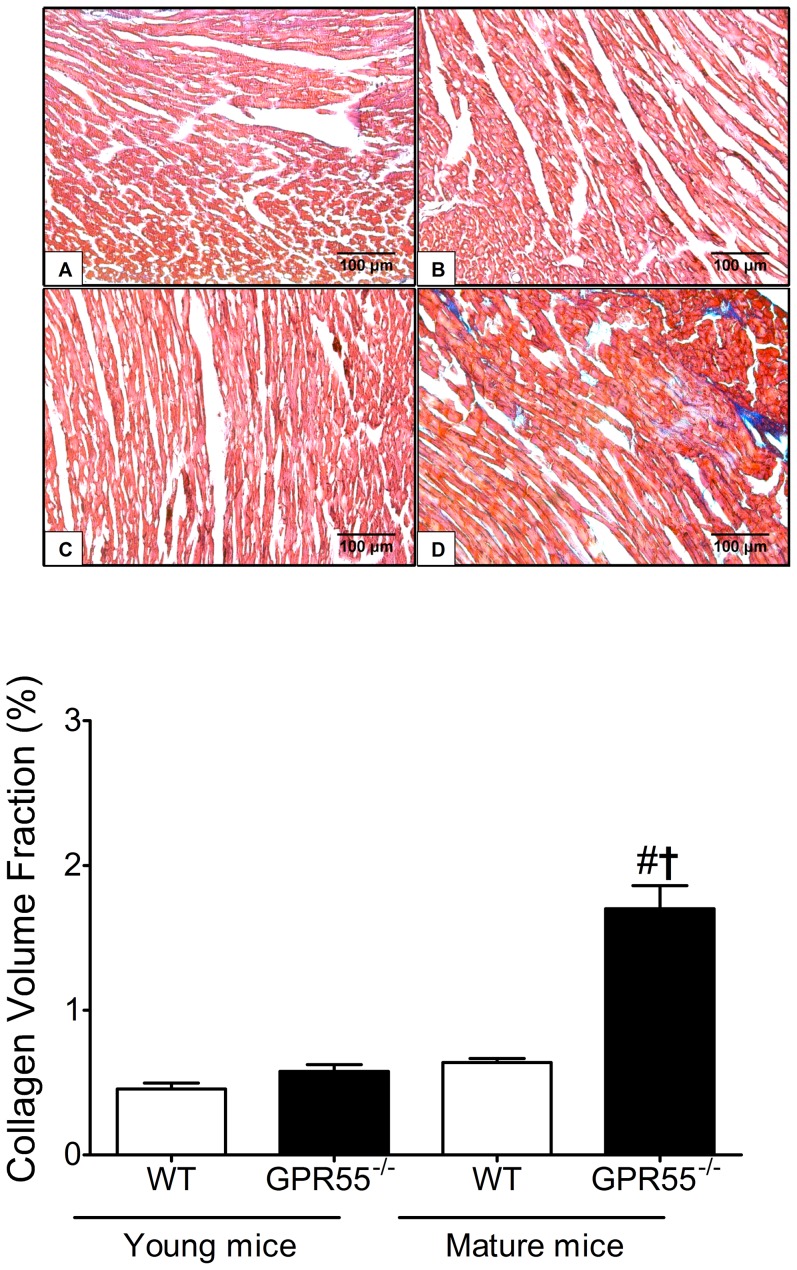
Influence of GPR55 gene deletion on cardiac collagen deposition. Representative photomicrographs (×400) demonstrating cardiac collagen deposition in young WT (A), young GPR55^−/−^ (B), mature WT (C), and mature GPR55^−/−^ (D) mice. Collagen deposition was significantly increased in the left ventricle of mature GPR55^−/−^ mice (E). Data is expressed as mean±s.e.m. (n = 14–15) #P<0.05 vs. GPR55^−/−^ (Young); †P<0.05 vs. WT (Mature).

Morphological measurements of cardiac dimensions revealed several significant age-related differences in WT mice. In particular, heart weight∶body weight ratio (HW∶BW; mg/g), cardiomyocyte cross-sectional area (CSA), and left ventricular (LV) wall thickness were all significantly increased in 8 month old WT mice compared to 10 week old WT mice (all *P*<0.05; Table 2). In contrast, HW∶BW, LV wall thickness, and nuclei number were all significantly decreased in the mature GPR55^−/−^ mice compared to age-matched WT mice (both *P*<0.05) and CSA was not significantly altered in comparison to young GPR55^−/−^ mice (Table 2). Furthermore, mature GPR55^−/−^ mice were characterised by significant increases in both interstitial and perivascular cardiac collagen deposition when compared to both the younger knockout mice and age-matched WT controls (*P*<0.05; Figure 4). While the latter may suggest significant ventricular remodelling (at least at the level of cardiac extracellular matrix composition), as this cardiac ‘fibrosis’ was not coupled with a significant upward shift in the EDPVR slope (Figure 2C) it seems unlikely that this ventricular remodelling influenced LV compliance and/or diastolic function in these mice. Finally, right ventricular wall thickness, interventricular septal wall thickness, and LV chamber area were all unchanged between all four groups of WT and GPR55^−/−^ mice (Table 2).

**Table 2:** Measurement of ventricular dimensions in WT and GPR55^−/−^ mice.

	Young Mice (10 week old)		Mature Mice (8 month old)	
	WT (n = 15)	GPR55^−/−^ (n = 15)	WT (n = 14)	GPR55^−/−^ (n = 14)
HW∶BW (mg/g)	3.07±0.06	3.19±0.1	3.98±0.06*	3.59±0.02†
LV Wall Thickness (mm)	1.68±0.04	1.79±0.11	1.92±0.05*	1.65±0.07†
RV Wall Thickness (mm)	0.39±0.02	0.38±0.04	0.36±0.04	0.40±0.01
Interventricular Septal Thickness (mm)	1.25±0.04	1.22±0.06	1.31±0.01	1.35±0.02
Chamber Area (% of LV)	13.9±0.9	15.5±1.3	12.8±0.7	13.2±0.8
Cardiomyocyte CSA (µm^2^)	89±9	94±8	145±5*	144±7
Nuclei number (nuclei/mm^2^)	6726±284	6456±169	6471±393	4983±405# †

Ventricular dimensions did not differ significantly between young WT and GPR55^−/−^ mice, however mature GPR55^−/−^ mice were characterised by significant myocardial remodelling; including a reduction in left ventricular (LV) free wall thickness, myocardial nuclei number, and HW∶BW, and increased collagen deposition. Data is expressed as mean±s.e.m. (n = 14–15).

*P<0.05 vs. WT (Young);

# P<0.05 vs. GPR55^−/−^ (Young);

† P<0.05 vs. WT (Mature).

### Effect of GPR55 gene deletion on contractile reserve in young and aged mice

Contractile reserve, assessed by the change from baseline cardiac function in response to the α_1_/β_1_-adrenoceptor agonist dobutamine, was significantly attenuated in both young and mature GPR55^−/−^ mice (Table 3). In young GPR55^−/−^ mice, the dobutamine induced increase in d*P*/d*t*_max_ was significantly attenuated compared to WT mice with a resultant reduction in the change in SV and consequently CO and EF (all *P*<0.01; Table 3). In addition, adrenoceptor mediated changes in d*P*/d*t*_min_ were also significantly decreased in the young GPR55^−/−^ mice (*P*<0.01; Table 3), suggesting an impaired lusitropic effect of dobutamine. The mature GPR55^−/−^ mice were also characterised by a decreased response to dobutamine in terms of increases in SV, CO, EF and d*P*/d*t*_max_ when compared to age-matched controls (*P*<0.001; Table 3). These changes occurred concomitant with a somewhat preserved lusitropic effect in terms of the rate of relaxation (i.e. d*P*/d*t*_min_), however EDV did not increase to a similar extent as that seen in the control group (*P*<0.001; Table 3). While mature GPR55^−/−^ were characterised by reduced contractile reserve, this was no worse than that observed in young GPR55^−/−^ mice.

**Table 3:** Effect of GPR55 gene deletion on contractile reserve in young (10 week old) and mature (8 month old) mice.

Δ from baseline	10 week old Mice		8 month old Mice	
	WT (n = 15)	GPR55^−/−^ (n = 15)	WT (n = 14)	GPR55^−/−^ (n = 14)
HR (BPM)	111±4	95±4*	94±10	101±8
ESP (mmHg)	19±5	2±3*	15±2	8±5
EDP (mmHg)	0.9±0.5	−0.4±0.3	0.1±0.3	−0.2±0.3
ESV (µL)	−1.3±0.6	−2.5±0.8	−3.8±0.9	−0.4±0.3†
EDV (µL)	3.7±0.6	−0.1±0.9	5.5±1.3	1.6±0.6†
SV (µL)	5.2±0.7	1.4±0.4*	8.9±0.9*	2.8±0.5†
SW (mmHg*µL)	571±71	98±40*	934±104*	294±89†
CO (µL/min)	2949±354	1810±245*	5893±443*	1541±278†
*E*_a_ (mmHg/µL)	−2±0.5	−1.2±0.3	−0.9±0.2	−2.5±1.4
EF (%)	14±2	8±2*	14±1	7±1†
d*P*/dt_max_ (mmHg/s)	6705±698	2098±306*	5934±681	2586±738†
d*P*/dt_min_ (mmHg/s)	−2389±489	−838±424*	−2384±247	−1233±529

Contractile reserve, assessed by the change from baseline cardiac function in response to the α_1_/β_1_-adrenoceptor agonist dobutamine, was significantly attenuated in both young and mature mice with a gene deletion for GPR55. Data is expressed as mean±s.e.m. (n = 14–15).

*P<0.05 vs. WT (Young);

† P<0.05 vs. WT (Mature).

## Discussion

Our findings demonstrate that genetic deletion of GPR55 in mice leads to the development of cardiac dysfunction with age and cardiac decompensation in response to adrenoceptor stimulation. While basal cardiac function was unaffected in young mice with a genetic deletion for GPR55, mature GPR55^−/−^ mice, which would still be considered young adult mice and unlikely to be affected by senescent heart dysfunction [20], were characterised by significantly comprised systolic function. In particular, both load-independent (ESPVR & *E*_max_) and load-dependent (ejection fraction) indices of systolic function are significantly decreased in the mature GPR55^−/−^ mice. Furthermore, mature GPR55^−/−^ mice were also characterised by significant myocardial remodelling; including reductions in left ventricular free wall thickness, HW∶BW and ventricular cell number, and increased collagen deposition. Taken together, these changes are indicative of the presence of some form of cardiomyopathy (an all-encompassing term referring to alterations in both cardiac structure and function, that lead to a deterioration in cardiac function and ultimately heart failure), and possibly one that possesses several of the features of dilated cardiomyopathy (DCM) i.e. LV wall thinning, comprised systolic function, and reduced contractile reserve.

Possible explanations for the emerging systolic dysfunction observed in the mature GPR55^−/−^ mice may include either impaired Ca^2+^ signalling in cardiomyocytes and/or altered central control of cardiac function. Studies have demonstrated GPR55 induced elevations in intracellular Ca^2+^ in both endothelial cells [21], and more recently cardiomyocytes [22]. In the latter study, Yu et al. [22] demonstrated that LPI applied both extra- and intracellularly induced elevations in [Ca^2+^]_i_ in a GPR55 dependent manner. Furthermore, these findings led them to suggest that the receptor was expressed both at the sarcolemma and endo-lysosomal compartment, which may explain the extensive expression of GPR55 observed in ventricular tissue in the present study. As the previous study has demonstrated a role for GPR55 in Ca^2+^ signalling in the cardiomyocyte [22], it is possible that GPR55 gene deletion could adversely affect excitation-contraction coupling in the cardiomyocyte, and consequently the contractile ability of both the cell and myocardium as a whole. However, as none of the Ca^2+^ dependent indices of contractility (d*P*/dt_max_, ESPVR or *E*_max_) differed between young GPR55^−/−^ and WT mice this seems unlikely.

An alternative explanation may be an affect on the central control of cardiac contractility. It is well established that in the early stages of systolic dysfunction, compensatory mechanisms are initiated to maintain systolic function and meet metabolic demands, including sympathoexcitation (reviewed by [23]). In particular, catecholamines acting on β_1_-adrenoceptors on pacemaker cells of the sinoatrial node, serve to increase action potential firing rate and induce a positive chronotropic response [24], [25]. In the present study, mature GPR55^−/−^ mice were not characterised by positive chronotropy (in an attempt to maintain systolic function), which may suggest impaired sympathetic control of the myocardium. In support of this, previous studies have demonstrated that activation of GPR55 leads to both increased excitability of dorsal root ganglion neurons [4], and enhanced pre-synaptic signalling in the hippocampus [26]. Although GPR55 expression in the nucleus tractus solitarius has yet to be demonstrated, it is possible that GPR55 may have a role in the regulation of synaptic transmission between preganglionic and postganglionic sympathetic efferents, and thus deletion of this GPCR may adversely affect sympathetic outflow. However, as basal systolic dysfunction appeared to be due to a chronic effect of GPR55 gene deletion (i.e. only evident at 8 months) and associated with significant ventricular remodelling it seems unlikely that GPR55 has a direct role in the control of cardiac function.

In the absence of a direct role for GPR55 in the control of cardiac contractility it is possible that this GPCR regulates the activity of another cardiac receptor responsible for regulating systolic function. Cardiac adrenoceptors, and β-adrenoceptors in particular, are the predominant GPCR in the heart and the chief modulators of both cardiac chronotropy and inotropy (reviewed by [27]). In the present study, all GPR55^−/−^ mice exhibited significantly attenuated positive inotropic responses to (±)-dobutamine (an agonist which directly stimulates cardiac adrenoceptors) when compared to WT mice, suggesting a pivotal role for GPR55 in the regulation of adrenoceptor activity in the myocardium. This proposed maladaptive adrenergic signalling may in part explain the progressive cardiac dysfunction associated with these GPR55^−/−^ mice. Accumulating evidence has shown that chronic stimulation of cardiac β-adrenoceptors activation leads to receptor phosphorylation via GPCR kinases i.e. βARK1 (desensitization), subsequent internalization of desensitized receptors via β-arrestin (downregulation), a loss of β-adrenoceptor mediated signalling, and finally the development of systolic heart failure (reviewed by [28]). Indeed, preservation of β-adrenergic signalling, via gene delivery of a βARK1 inhibitor, can reverse and/or prevent the development of cardiac dysfunction [29], [30]. Thus it's possible that the systolic dysfunction evident in the mature GPR55^−/−^ mice may be due to the progressive loss of cardiac adrenoceptors. In line with this it might have been expected that the impaired positive inotropy to dobutamine observed in the young GPR55^−/−^ mice would be further attenuated in the mature knockout mice, however this was not the case.

A possible explanation for this may involve α_1_-adrenoceptors, as although β_1_-adrenoceptors are thought to be primarily responsible for catecholamine induced increases in cardiomyocyte contractility, α_1_-adrenoceptors have been shown to induce cardiac contraction [31], [32], [33]. Furthermore, they have previously been shown to mediate part of the (±)-dobutamine induced positive inotropy in the rodent heart [34]. Although α_1_-adrenoceptor expression in healthy murine and human hearts is considerably less than that of the β-adrenoceptor subtypes [35], β-adrenoceptors are downregulated in heart failure whereas α_1_-adrenoceptors are not [36], [37]. Thus α_1_-adrenoceptor-mediated responses may contribute substantially to the compensatory positive inotropy in failing hearts.

In addition to altered cardiac function, mature GPR55^−/−^ mice were also characterised by significant ventricular remodelling including decreased HW∶BW, a thinning of the LV wall, a reduction in myocardial cell number, and increased collagen deposition. While there is currently no direct evidence for a functional role for GPR55 in the control of fibroblast activity, a recent study has demonstrated that this receptor is expressed on cells, which likely include fibroblasts, in the adventitial layer of rodent vasculature [38], and that LPI is synthesised by transformed mouse BALB/3T3 fibroblasts [39], thus it is possible GPR55 may play a role in fibrogenesis. Indirectly, GPR55 may regulate fibrogenesis by altering the activity of another GPCR i.e. CB_1_, as it has recently been demonstrated that GPR55 can form a heteromer with CB_1_ allowing the former to alter the signalling mechanisms/activity of the latter, and vice versa [40]. Therefore it is possible that the profibrogenic effect of CB_1_, previously documented in an experimental model of doxorubicin-induced cardiomyopathy [41], may be unimpeded in the mature mice lacking GPR55 thus resulting in the development of mild cardiac fibrosis. Finally, if mature GPR55^−/−^ mice are in fact characterised by increased cardiac α_1_-adrenoceptor activity (as discussed in the previous section) then this may account for the increased myocardial collagen deposition as cardiac fibrosis has previously been demonstrated in mice overexpressing α_1_-adrenoceptors [42].

The observation that mature GPR55^−/−^ mice are characterised by cardiac fibrosis seems somewhat incongruous with the significant reductions in both HW∶BW and LV wall thickness (indicative of a ‘lighter’ heart) observed in these animals. Therefore rather then increased fibrogenesis being the culprit for the increased cardiac collagen deposition, the latter may simply be ‘increased’ in the face of increased cell death/loss from the heart. In the present study, the left ventricles of mature GPR55^−/−^ were characterised by a signficant reduction in stained nuclei indicating an increase in myocardial cell loss. However, as this data was acquired from H&E stained tissue, which is not specific for cardiomyocytes, we cannot conclusively say that all of the cell loss was due to cardiomyocyte apoptosis and additional studies are required. Previous work has suggested both anti-inflammatory [43] and anti-oxidant [44] roles for GPR55, thus loss of the receptor may lead to a chronic upregulation of both inflammation and oxidative stress in the mature GPR55^−/−^, both of which are chief instigators of cardiomyocyte cell death. However as the present study did not examine either the inflammatory or oxidative status of these animals these proposed mechanisms remain to be confirmed.

Exactly how the deletion of the GPR55 gene affects cardiac adrenoceptor signalling/function in the present study is unclear. However, accumulating evidence has demonstrated that co-localised GPCRs, not limited to adrenoceptor subtypes alone [45], [46], [47], but adrenoceptors and other GPCRs, can interact and regulate surface expression of each other via a process termed dimerization (reviewed by [48]). In particular, data from isolated ventricular cardiomyocytes has demonstrated cross-regulation between adrenergic and adenosinergic receptors, where stimulation of one inhibited the activity of the other and *vice versa* [49]. Furthermore, as previously discussed GPR55 can form heteromers with CB_1_ enabling both GPCR's to alter the signalling mechanisms/activity of the other [40]. In heart failure, the crosstalk between α-adrenoceptors and β-adrenoceptors is well established, in that expression of the former is elevated in the response to the downregulation of the latter as a means of sustaining positive inotropism of the contractile apparatus [50], [51]. While studies have yet to demonstrate co-expression of GPR55 and adrenoceptors within the same cardiomyocytes (as was demonstrated in murine vascular cells [38]), it is possible that there is some level of co-localisation in the myocardium that may facilitate crosstalk between these GPCRs influencing their function/expression, although this requires investigation. Finally, as the present study only examined the impact of GPR55 gene deletion on the function of adrenoceptors, we cannot rule out the possibilty that other GPCR's are similarly adversely affected. Furthermore, rather than GPR55 having a direct effect in terms of modulating other GPCR's function it is possible that the absence of this receptor may result in a more generalised adverse effect i.e. defective G protein-coupled signalling, culminating in the dysfunction of numerous GPCRs particularly those involved in stress-sensitive pathways. Thus the effect of GPR55 gene deletion on other GPCRs capable of inducing both inotropic and chronotropic responses should also be investigated in the future.

## Conclusions

The present study has demonstrated that mature GPR55^−/−^ mice are characterised by a progressive ventricular dysfunction. This intrinsic inability of the heart to maintain systolic function appears to be due to maladaptive adrenergic signalling, which may suggest some interplay/crosstalk between GPR55 and adrenoceptors and a possible role for GPR55 in the pathogenesis and/or progression of heart failure.
